# Understanding the Impact of Scale Height on the Kinetics and Kinematics of Dogs in Working Trials

**DOI:** 10.3389/fvets.2021.742068

**Published:** 2022-02-16

**Authors:** Anne Carter, Jacqueline Boyd, Ellen Williams

**Affiliations:** ^1^School of Animal, Rural and Environmental Sciences, Nottingham Trent University, Nottinghamshire, United Kingdom; ^2^Department of Animal Health, Behavior and Welfare, Harper Adams University, Newport, United Kingdom

**Keywords:** peak vertical landing force, working trials, canine, biomechanics, joint angulation

## Abstract

Working trials is a canine discipline that originated from police and military dog work. One aspect of working trials competition is for a dog to “scale” a 6ft high wooden wall. Concern has been raised in other canine disciplines that landing forces after traversing jumps may lead to soft tissue injuries. There is a paucity of research into the impact of scale height on peak vertical landing force (PVF) in dogs participating in working trials. The aim of this work was to determine whether an alteration in scale height impacts PVF and apparent joint angulation on landing. Twenty-one dogs who regularly competed in working trials traversed the scale at three different heights; 6ft (full height), 5.5ft and 5ft. Changes in PVF, apparent carpal and shoulder joint angulation and duration of landing were analyzed using general linear mixed models. Dogs weighing >25 kg had greater PVF at 6ft than at 5ft (*p* < 0.05). There was no effect of scale height on PVF in dogs <25kg. Duration of landing was longer at 5ft than 5.5ft (*p* < 0.001) and 6ft (*p* < 0.001). Apparent carpus angle on landing was smaller at 6ft than 5ft (*p* < 0.05) and 5.5ft (*p* < 0.05) for dogs <25 kg. Apparent carpus angle on landing did not differ at any height for dogs >25 kg (*p* > 0.05). Apparent shoulder angle was not affected by scale height for any dogs (*p* > 0.05). There was considerable variation in the study population, but this research indicates that when the scale height was lowered to 5.5ft dogs had reduced PVF and less compressed joint angles on landing. When the scale height was lowered to 5ft they altered their traversing style and greater compression and increased PVF was seen. Evidence-based approaches to canine working trials are important to ensure minimum impacts on physical health and welfare of participating dogs, in terms of risk of injury in both competition and training. Based on these findings it is recommended that the maximum height of the scale is reviewed for training and competitive purposes, to ensure minimal impacts on the health of competing dogs, while maintaining the level of competitive challenge.

## Introduction

Working trials is a canine discipline originating in the 1920's from police and military dog work and has seen little modification in format since the 1960's. The discipline is split into three components, scent work, agility [clearing a 6ft scale (wall), 9ft long jump and a 3ft hurdle under control], and obedience tasks (1). The scale obstacle in the agility component of working trials is considered particularly physically demanding for the dogs, with a requirement for the dog to jump from a static start on the ground to ‘scale’ the obstacle, landing in a controlled manner, before returning over the scale. Whilst the scale obstacle originated from police dog training, the chosen maximum height for specific competitive levels (dogs exceeding 15 inches at the shoulder) is currently an arbitrary measurement of 6ft (2).

Concern has been raised about potential injury risk to dogs participating in other canine disciplines such as agility, where dogs traverse a series of jumps and other obstacles as a test of speed and athletic ability. Landing forces experienced while participating in agility have been postulated to potentially result in soft tissue injuries, notably to the back and shoulder (3–6). Studies have explored the effect of jump height (7), and distance between jump obstacles (8, 9) on the kinematics, landing forces and apparent joint angles of participating dogs. As obstacle height is increased, peak landing forces in dogs also increases (10). Both horses (11) and dogs (7) alter their apparent joint angulation as hurdle height is altered. In addition, horses have been shown to alter joint angles at take-off, suspension and landing based on both jump height and jump type (11–13). Furthermore, Birch et al. (7) also demonstrated that when dogs were asked to jump an upright hurdle that was >76% of height to their wither height, their kinematics demonstrated alterations. It thus appears that dogs and horses significantly alter their kinematics based on obstacle height.

Wider canine kinematic research suggests that peak landing force is higher when landing over an upright hurdle than running or landing over a long jump for dogs (14). On landing following a simulated jump from a car boot, peak ground force increased as the height of the platform increased (15). Whilst the working trials scale is an “up and over” obstacle, the height results in the dogs reaching the top before coming down on the other side, with a momentary pause on the top of the scale as they maneuver over the top, rather than clearing the obstacle as they would a hurdle. In cats, peak vertical force increased as the height to a landing surface was increased (16). Higher peak vertical landing forces (PVF) may increase forelimb and shoulder injury risk in dogs (3–6). Yanoff et al. (10) highlighted body mass as a significant factor in relation to peak vertical ground force. Whilst the assessment of standard gait of dogs did not vary according to body weight, the loading of dog limbs on landing may be impacted by the body weight of the dogs.

The height of the scale obstacle in working trial competitions is based on arbitrary measurements, with the maximum height for dogs > 15 inches at the shoulder, being 6ft high. There is a paucity of research on the impact of scale height on PVF and apparent joint angulation of dogs on landing, which may have ramifications for the physical health of dogs participating in this discipline. The aim of this study was thus to determine whether an alteration in scale height impacts peak vertical landing force and apparent joint angles on landing in experienced dogs routinely training and competing in working trials.

## Materials and Methods

### Ethics Statement

All research protocols were approved by Nottingham Trent University, School of Animal, Rural and Environmental Sciences School Ethics Group (reference number ARE192042).

### Study Population

Dogs were recruited opportunistically from the population of handlers and dogs regularly competing in working trials in the UK. All dogs had trained or competed in working trials for at least 12 months to minimize the effect of naive or inexperienced dogs. Dogs were therefore experienced in clearing the scale obstacle at the maximum competitive height.

Twenty-one dogs (15 male, 6 female) were recruited to the study from five breeds/types (identified by handlers): border collie/working sheep dog (*n* = 10), golden retriever (*n* = 1), German shepherd/malinois (*n* = 4), Labrador retriever (*n* = 5), spaniel cross Labrador (*n* = 1). Median age of dogs was 4.5 years (range 2–8 years). Dogs <25 kg (*n* = 12) had a mean ± SD bodyweight of 21.5 ± 2.4 kg, dogs >25 kg (*n* = 9) had a bodyweight of 29.2 ± 4.3 kg. Demographic details of the study population are provided in Table 1. All dogs were declared as physically fit enough to undertake this study by their handlers, this included being free from any current injuries. Signed consent was given for their participation in the study.

**Table 1:** Demographics of participating dogs.

**Dog**	**Sex**	**Breed/type**	**Age (yr)**	**Height to withers (cm)**	**Weight (kg)**	Number of scale completions^*^		
						**5ft**	**5.5ft**	**6ft**
1	M	Working sheep dog	8	59.0	23.7	3	3	3
2	M	Border collie	4	49.5	21.7	3	4	3
3	M	Labrador retriever	5	57.0	27.7	4	3	3
4	F	Labrador retriever	3	51.0	21.0	3	3	4
5	M	Working sheep dog	3	56.5	24.1	7	3	3
6	M	Border collie	7	56.5	23.3	5	3	3
7	M	Working sheep dog	8	53.0	17.8	3	3	3
8	M	German shepherd	6	65.0	40.0	1	0	0
9	F	Labrador retriever	5	55.0	24.6	4	4	4
10	F	Border collie	2	48.3	17.2	3	3	3
11	M	Labrador retriever	5	57.0	31.3	3	3	3
12	M	Labrador retriever	4	57.0	25.8	4	3	3
13	M	Spaniel/Labrador retriever cross	6	47.0	23.2	3	3	3
14	M	Border collie	3	56.0	25.2	4	3	3
15	F	German shepherd	5	No data^**^	30.2	3	3	3
16	F	Working sheep dog	3	52.0	18.6	3	3	3
17	M	Border collie	2	55.0	21.3	3	6	6
18	F	German shepherd	3	56.5	26.3	3	6	4
19	M	Malinois	No data^**^	54.0	26.6	3	3	3
20	M	Working sheep dog	7	52.0	21.7	3	3	3
21	M	Working golden retriever	4	57.0	29.8	4	3	3

* *Dogs traversed the scale height until they were considered to have landed successfully on the pressure mat three times (visual assessment from the project team). Continued traversing of the scale to achieve three successful landings on the pressure mat was at the discretion of the handler*.

** *Where no data was collected, this was due to omission or due to difficulty in measuring height*.

### Experimental Setup

**Figure 1:**
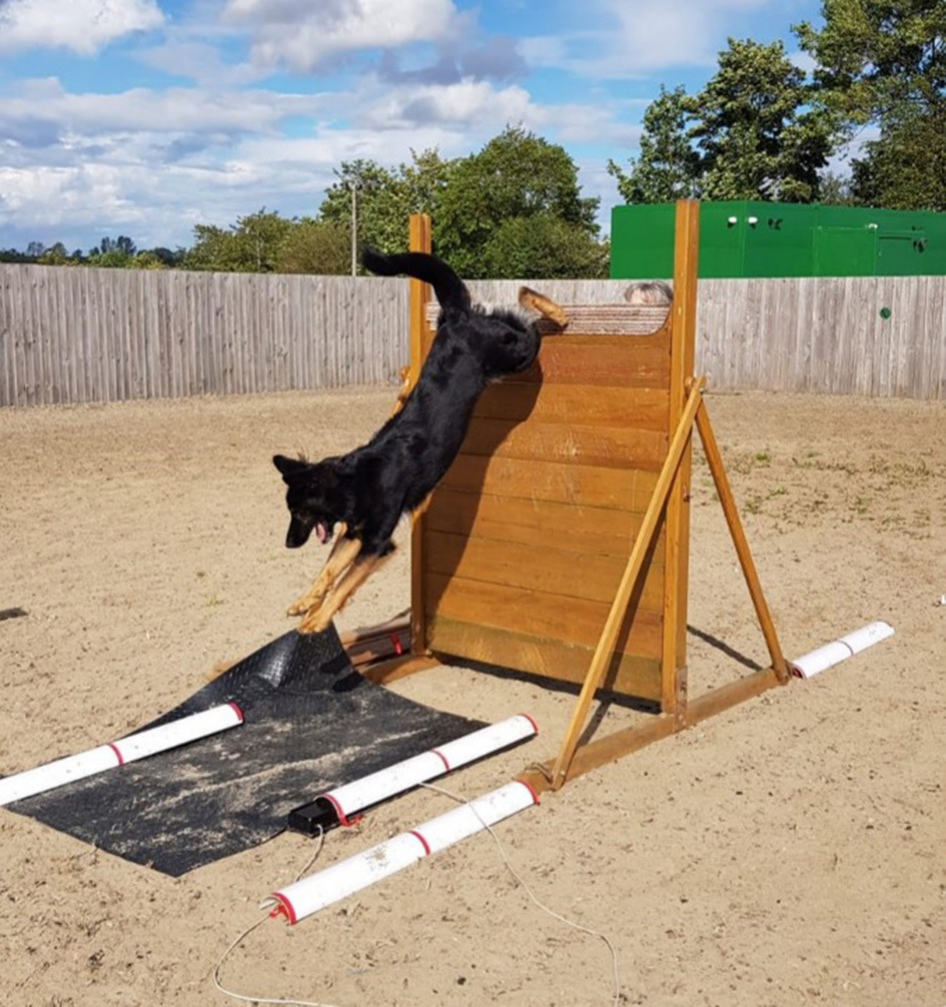
Scale study setup showing positioning of pressure sensing mat on landing side. The participating dog was traversing the scale at 5ft.

The study was carried out in a fenced outdoor equestrian arena with a fiber sand surface. The handlers prepared their dogs, as they would prior to the scale element of the working trials competition. This also allowed the dogs to acclimatize to the research environment and equipment. The study examined dogs traversing the scale at three different heights. 6ft (1.83 m) (the current maximum KC height in competition for dogs exceeding 15 inches (38.1 cm) at the shoulder), 5.5ft (1.71 m) and 5ft (1.52 m). This was the equivalent to removing one plank from the scale each time. Dogs were directed by their handler throughout the study. Dogs traversed the scale as they would do in normal training or competition and were asked to complete the scale exercise three times per height. Where dogs did not land fully on the pressure sensing equipment, they were requested to repeat the height to achieve three successful landings on the mat. The number of times each dog traversed the scale is included in Table 1. The order of the three heights was randomized. No time limit was put on completion of the obstacle; therefore, the owner could take breaks between attempts. If a dog failed to complete a scale, they were given one further attempt at that height, following a second failed attempt, the dog was withdrawn from the study. Dogs were withdrawn from the study at the owner's discretion. Dogs were filmed during the landing phase of their traversing of the scale using high-definition video cameras (JVC-GC PX10 HD, 300 fps) with lateral placement to the scale with a 1 m ground marker for reference (Figure 1).

#### Peak Vertical Landing Force

**Figure 2:**
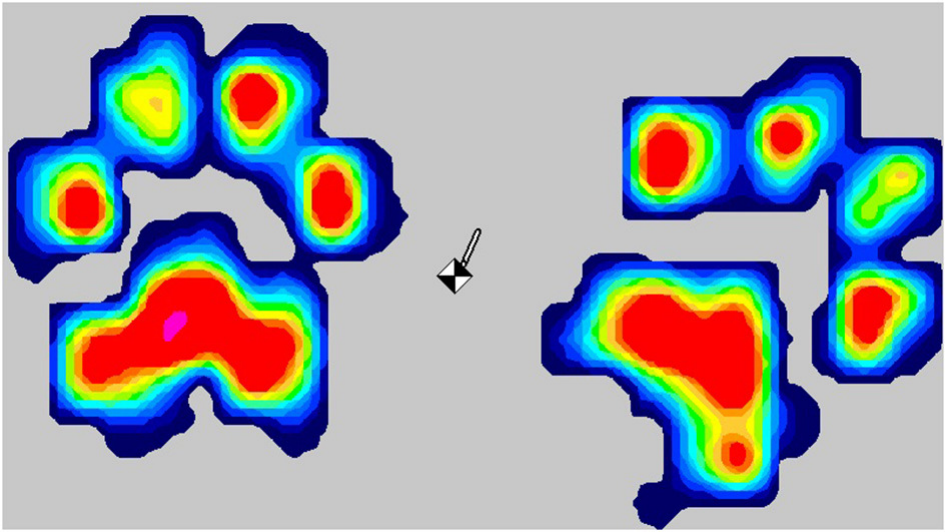
Tekscan “heatmat” visualization of landing force. Colors provide a visual representation of measured forces from low (blue) to high (red). The black and white symbol indicates center of gravity at the point of measurement.

A Tekscan walkway gait analysis system 3,150 pressure (sensing area of 0.87 × 0.37 m, maximum 100 Hz) (Tekscan) was placed at the landing point (Figure 1), covered by a thin rubber mat to standardize the landing surface. This was used to measure peak vertical force (pounds) on landing. Peak vertical force on landing across both front feet was measured using Matscan XL (Figure 2). If only one foot landed on the mat this replicate was discarded.

#### Apparent Joint Angles and Duration of Landing

**Figure 3:**
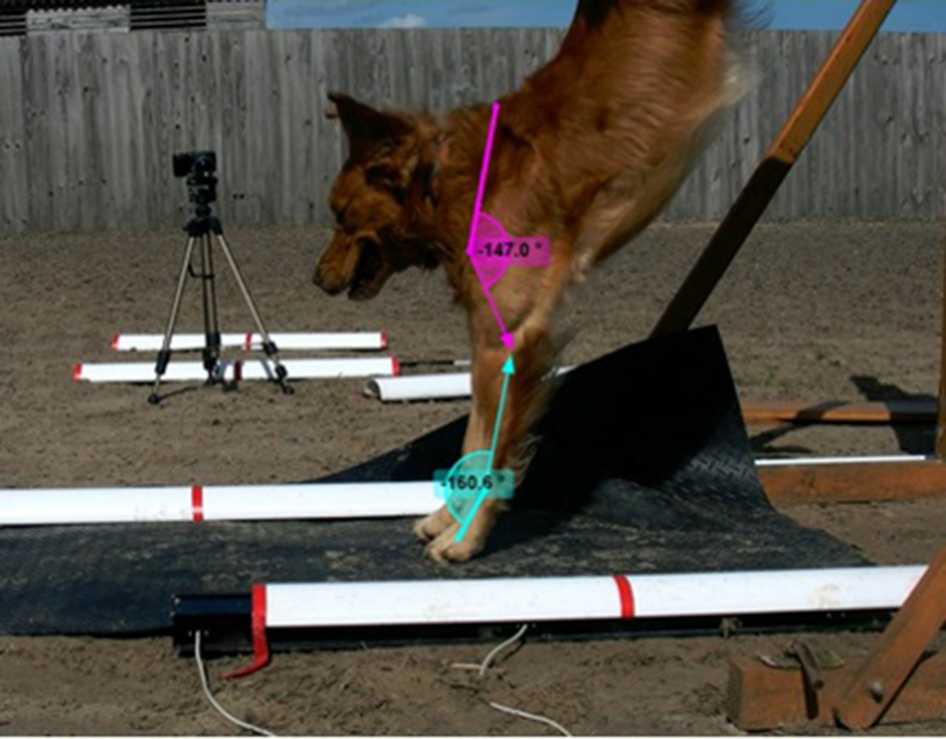
Apparent angles of the carpus and shoulder of dogs on landing, measured using Kinovea Version 0.9.3.

Apparent carpus and shoulder angles on landing and duration of landing were measured using Kinovea Version 0.9.3. Apparent angles of the carpus and the shoulder of dogs on landing (Figure 3) were measured on each video frame (30 fps) during the landing from the time the first front foot touched the floor to the time the first rear foot hit the floor. Measurements were taken using a markerless system [as per (17)]. The frame at which the dog had the minimum carpus angle was taken to be the lowest phase of the landing. Minimum carpus angle, shoulder at the lowest phase of the landing and minimum shoulder angles were used for analysis. Duration of landing was measured in seconds (using video frames).

### Data Analysis

General linear mixed models, with Tukey corrected *post-hoc* tests where appropriate, were used to investigate the impact of scale height (5ft, 5.5ft, 6ft) and dog weight (<25 kg, >25 kg), on PVF, minimum carpus and shoulder angles on landing and duration of landing (seconds). Five models were created: PVF (measured in pounds) as a function of body weight (PVF/kg), duration of landing (seconds), carpus angle at lowest phase of the landing (minimum carpus angle), shoulder angle at lowest phase of the landing and minimum shoulder angle. To prevent erroneous identification of PVF, individual jumps were only included in analysis if values were present for both front feet, to enable identification of the maximum PVF across both feet. Peak vertical landing force, landing duration and angles of interest were fitted as response variables. Scale height and dog weight were fitted as fixed effects. To control for replicates, dog was included as a random effect in each model. Data analysis was undertaken in R Studio (Version 4.0.3) (18) using packages “lme4” (19) and “emmeans” (20). Variance in PVF/kg, apparent angles on landing and landing duration between dogs <25 kg and >25 kg at the three scale heights (5ft, 5.5ft, 6ft) was assessed using a Levene's test using package “car” (21). Graphs were produced using package “ggplot2” (22). Model results are reported as model estimate (β_1_) ± SE. Significance values were set at *p* < 0.05 for all analysis.

## Results

### Peak Vertical Landing Force

**Figure 4:**
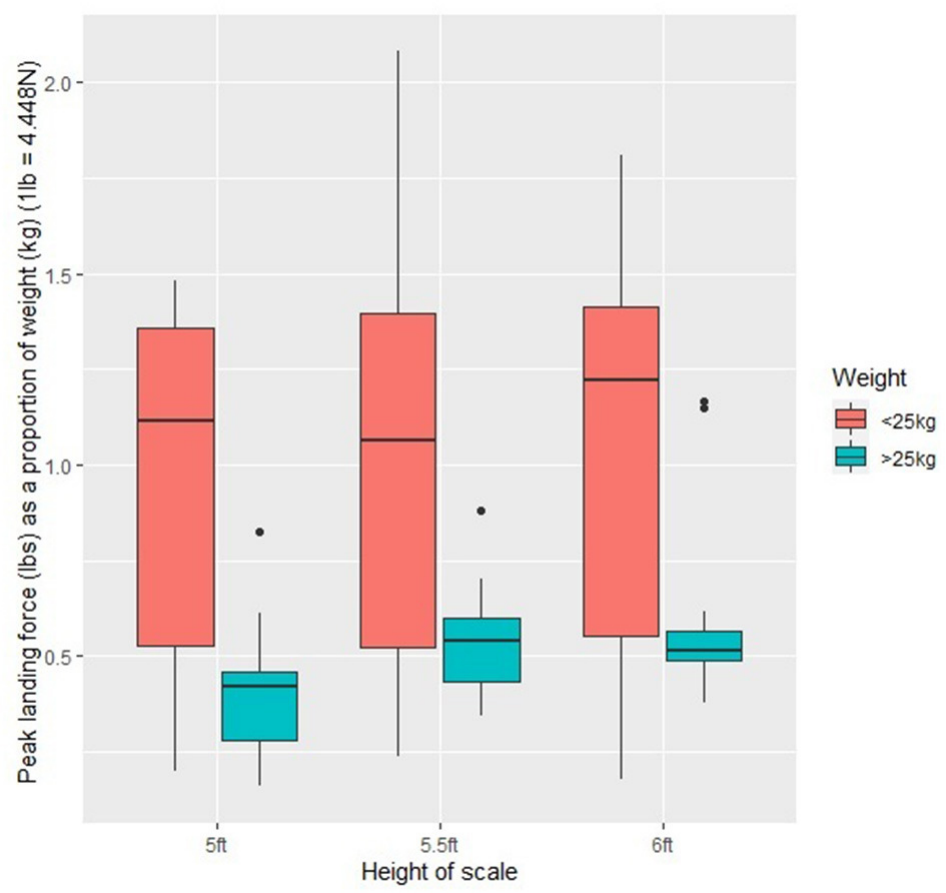
Peak vertical landing force (PVF, measured in pounds) as a proportion of body weight across all the study dogs (*n* = 20) at the three scale heights (5ft, 5.5ft, and 6ft). (N.B.1lb = 4.448N).

When the whole study population was considered there was no relationship between PVF (measured in pounds) as a proportion of dogs' bodyweight (PVF/kg) at the three scale heights (*p* > 0.05). When this was investigated in terms of weight categories, there was no significant difference in PVF/kg for dogs <25 kg at any of the scale heights (*p* > 0.05). Dogs >25 kg had significantly lower PVF/kg at 5ft than 6ft (−6.102 ± 1.92, *t* = −3.173, *p* = 0.02) but there was no difference between 5ft and 5.5ft or 5.5ft and 6ft (*p* > 0.05). PVF/kg varied across dogs. There was a trend toward lighter dogs (<25 kg) having a greater PVF/kg than dogs >25 kg (−7.423 ± 4.14, *Z* = −1.793, *p* = 0.07). There was greater variation in PVF/kg in dogs <25 kg (*F* = 10.165, *p* < 0.001) (Figure 4).

### Duration of Landing

**Figure 5:**
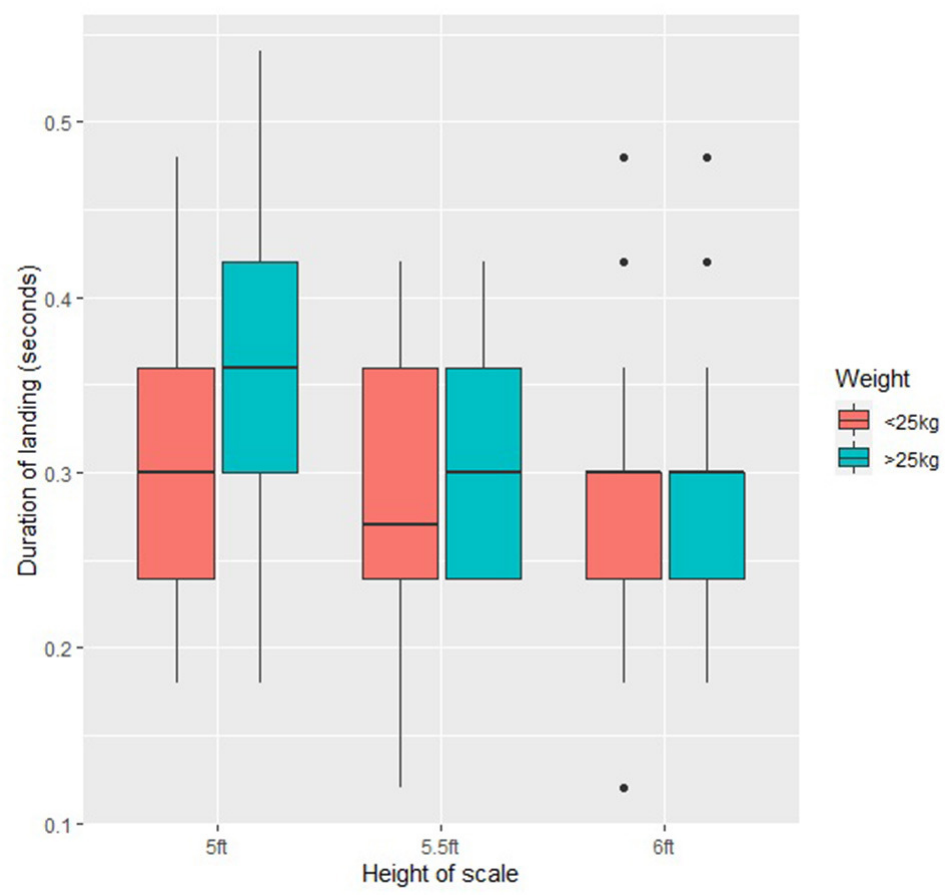
Mean duration of landing (seconds) across all the study dogs (*n* = 21) at the three scale heights (5ft, 5.5ft, and 6ft).

Across all of the study population, duration of landing was longer for dogs at 5ft (mean ± SD, 0.33 ± 0.09 s) than 5.5ft (0.29 ± 0.07 s) (−1.30 ± 0.35, *t* = −3.718, *p* < 0.001) and 6ft (0.29 ± 0.08 s) (−1.43 ± 0.35, *p* < 0.001). There was no significant difference in duration of landing between 5.5ft and 6ft (*p* > 0.05). This was then considered within the two weight categories. Duration of landing was longer for dogs <25 kg at 5ft (0.31 ± 0.09 s) than 5.5ft (0.28 ± 0.08 s) (1.3662 ± 0.466, *t* = 2.933, *p* < 0.05) (Figure 5). In dogs >25 kg landing duration was significantly longer at 5ft (0.35 ± 0.09 s) than 6ft (0.30 ± 0.07 s) (−1.6814 ± 0.541, *t* = −3.105, *p* < 0.05). There was no significant difference in variation in duration of landing in dogs <25 kg and >25 kg (*p* > 0.05).

### Apparent Angulation of Carpus and Shoulder

**Figure 6:**
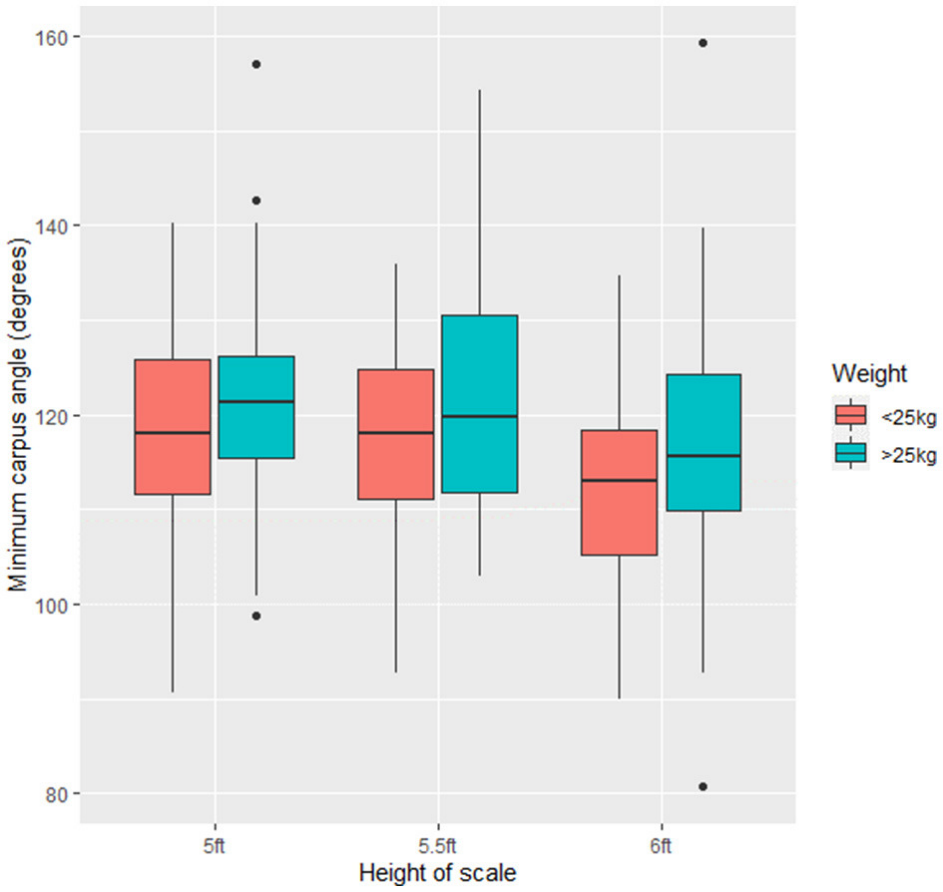
Minimum apparent carpus angle across all the study dogs (*n* = 21) at the three scale heights (5ft, 5.5ft, and 6ft).

The apparent carpus angle on landing was significantly smaller at 6ft than 5ft (5.590 ± 1.80, *t* = 3.104, *p* < 0.05) and mid height (5.5ft) (5.289 ± 1.80, *t* = 2.945, *p* < 0.05) for dogs <25 kg (Figure 6). There was no significant difference in apparent carpus angle on landing at any height for dogs >25 kg (*p* > 0.05). Neither minimum apparent shoulder angle nor apparent shoulder angle at the lowest phase of the jump was affected by scale height in either dogs weighing <25 kg or dogs >25 kg (*p* > 0.05). There was no significant difference in variation in apparent joint angles on landing for dogs <25 kg and >25 kg (*p* > 0.05).

## Discussion

Working trials is a canine discipline that involves participating dogs traversing an upright wooden “scale” as an integral part of the agility test of the activity. Within specific competitive levels the maximum height (6ft) of the working trials scale is based on arbitrary measurements with no scientific research to inform the suitability of the height for participating dogs. There is a paucity of research on the impact of scale height on landing forces and apparent joint angulation of dogs on landing after traversing the scale. This contrasts with the discipline of dog agility, where research has identified specific kinematic and ground reactive force alterations in participating dogs (8, 9, 14, 23). The height of scale used in working dog trials may thus have ramifications for physical health of dogs participating in this discipline.

The aim of this study was to determine whether an alteration in scale height alters PVF and apparent carpal and shoulder angles on landing. This was assessed in in dogs routinely training and competing in working trials. To determine the impact of dog weight on carpal and shoulder angles on landing, dog weight was investigated in terms of <25 and >25 kg. There was significantly different variation in dogs <25 and >25 kg and data indicates that dogs of different bodyweights were taking different approaches to landing after traversing the scale.

### Peak Vertical Landing Force

Cats jumping from a flat surface (16) and dogs jumping from car boots (15) show increased PVF when landing from greater heights. This was partially replicated in this study, with dogs >25 kg showing significantly greater PVF at 6ft than 5ft. However, dogs <25 kg showed no significant changes in PVF at any of the heights. Pfau et al. (14) highlighted very high peak vertical force in the forelimbs of dogs (25 N/kg per foot) when landing from hurdle jumps at speed. This was not observed in the present study, although Pfau and colleagues examined border collie dogs of up to 19 kg, which was the weight category in which we found greatest variation despite less variability in bodyweight (dogs <25 kg 21.5 ± 2.4 kg, dogs >25 kg 29.2 ± 4.3 kg). The greatest PVF recorded was in dogs <25 kg at the highest (6ft; 8 N/kg) and middle (5.5ft; 9.3 N/kg) scale heights. However, considerable variation was observed in the study population. It is also important to highlight that dog agility involves dogs negotiating hurdles at speed and velocity affects limb dynamics in agility dogs (23). Working trials obstacles are traversed with significantly less emphasis on speed. This may permit more dynamic kinematic adaptation by participating dogs, which is supported by the altered apparent landing angles observed in this study.

### Apparent Joint Angulation on Landing

In dogs of a lighter bodyweight (<25 kg) there was significantly more apparent compression on the carpal joint at 6ft than 5ft and 5.5ft. However, there was no significant difference in landing force across any of the three investigated heights, which suggests that dogs <25 kg are absorbing the landing force through their carpal joint. It is thus possible that dogs are “shock absorbing” the force of their landing though their joints. Research exploring limb dynamics in beginner vs. advanced agility dogs showed increased limb compression during the stance phase of landing in beginner dogs compared to advanced dogs (23). This suggests that experience and training may influence how dogs traverse and respond to specific equipment. Miro et al. (24) similarly demonstrated that experience affected the kinematics of how agility dogs traversed a hurdle. In the present study, median age of dogs was 4.5 years (range 2–8 years). All dogs and handers were experienced participants; dogs had been training in working trials for a minimum of 12 months. It is likely that the study dogs have developed the ability to dynamically respond to differential scale heights through training and experience. Future research to explore the impact of training and experience on kinematics of dogs in working trials is recommended, to further advance knowledge in this area and support the development of evidence-based guidelines in this discipline.

Research has indicated that both dogs (8, 9, 14) and horses (25) show variation in joint angles upon landing, and similar findings were found in this research. Dogs of >25 kg had a greater landing force at 6ft than 5ft but no significant difference was observed between 5.5 and 6ft. Although not significant, descriptive statistics indicate larger apparent carpal and shoulder angles (indicative of reduced compression on landing) at 5.5ft as compared to both 5ft and 6ft. Observations of study dogs during the trials indicated that they altered their style when traversing the 5ft scale, with some individuals trying to “jump” rather than “scale” the obstacle. It is thus possible that there are benefits to dogs in reducing the scale to 5.5ft; evidenced by reduced compression on landing, but that when the scale is reduced to 5ft these benefits are lost as the obstacle might be tackled in a different manner, thus resulting in potential impacts as highlighted in the canine agility literature. This is also significant from a competitive perspective where a level of challenge is typically required.

The observed alteration in scale traversing style was reflected in the duration of landing. Landing duration was measured from when the first front foot hit the pressure mat until the first back foot hit the mat. Dogs >25 kg showed no variation in landing duration at any of the heights, however in dogs <25 kg landing duration was longer at the 5ft (mean ± SD seconds, 0.31 ± 0.09) scale than 5.5ft (0.29 ± 0.08) or 6ft (0.28 ± 0.09). Increased duration of landing contact may be due to dogs striding off the scale through a dynamic motion, rather than the more traditional stationary landing when they have “scaled” the obstacle and released themselves from the top. They are thus potentially traversing the lower height scale like a hurdle obstacle, rather than a scale.

### Limitations of the Research, Future Directions, and Recommendations for Working Trials

PVF measurements should take into account sampling frequency, which is affected by the sensing equipment used. The use of a force plate would have given a higher sampling frequency (up to 1,000 Hz) and a more accurate response (26), in addition to the capacity to measure mediolateral and craniocaudal forces. However, the field-based nature of the study limited the opportunity to use a force plate rather than a pressure mat to record PVF. This study focused on jumping down from an obstacle, therefore limiting the forward trajectory of the dogs and thus minimizing the impact of this limitation.

Dogs included in the study were representative of the breeds/types typically participating in UK working trials. However, numbers of individuals in terms of breed/type category were limited, which prevented breed/type-level analyses being undertaken. Due to this it was also not possible to differentiate beyond arbitrary weight categories. As significant differences were seen between dogs <25 and >25 kg and considerable variation was seen in dogs <25 kg, we strongly advocate undertaking such work in a wider study population, to determine the impact of greater variation of weight categories, and breed/type-level differences (27). There may be a requirement to consider breed/type and/or weight effect in dogs traversing the scale, to further understand individual participant effects. Indeed, there may already exist a level of “self-selection” in participating dogs, where those with a bodyweight significantly above or below an arbitrary threshold are less successful in competition. Further examination of the physical characteristics of participating dogs could further our understanding of key biological characteristics linked to success, in the same way as has been postulated for horses (25).

Evidence of shoulder injury has been reported in beginner agility dogs (3, 5, 6), however it is known that experience impacts kinematics in these dogs (23, 24). It is thus likely that injury reported in these studies is related to experience of the participants. It was beyond the scope of this study to investigate injuries in the study population, and no dogs involved in this research had any current injuries. However, establishing whether there are consistent joints in which injuries are occurring in the wider working dogs trial population would enable a greater understanding of whether there is any long-term impact on joint health, and how that may relate to participation in working trials. This is thus an area of research which we advocate being undertaken.

We recommend a review of the maximum scale height in working trials based on study findings and monitoring impacts on the wider working trials population, both in competition and in training. Reducing the scale height to 5.5ft is likely to reduce the PVF experienced by dogs with a bodyweight of >25 kg. In dogs with a bodyweight of <25 kg it may reduce the apparent compression of the carpal joint, whilst not leading to alterations in the way that dogs approach and traverse the obstacle. This could be of relevance in training and competitive situations. Competition may wish to retain the “challenge” of a higher scale, while training at a lower height of 5.5ft permits handlers and dogs to gain experience, with dogs experiencing reduced kinematic impact. Further reduction in scale height to 5ft has the potential to alter dog kinematics and thus lose any benefit in terms of reduced landing impact.

We also advocate for investigation of the impact of landing surface. Research has indicated that landing surface can alter landing and braking kinematics in horses (28). Working trial participants experience a range of surfaces both in training and in competition and so dogs may be landing on harder or softer landing surfaces. This could impact on PVF and apparent joint angulation, and thus is something that should be further investigated.

## Conclusion

Evidence-based approaches to canine working trials are important to ensure minimum impacts on physical health and welfare of participating dogs, in terms of risk of injury in both competition and training. This research indicated that a reduction in the height of the scale in working trials from 6ft to 5.5ft may have positive implications for longitudinal physical health of dogs. Reducing the height of the scale to 5.5ft led to reduced PVF in dogs >25 kg and reduced apparent compression of the carpal joint in dogs <25 kg, without altering the way that dogs tackled the obstacle. We thus recommend reviewing the frequency at which working trials dogs experience the maximum height of the scale in both training and competition, while also maintaining a level of competitive challenge. Further research is needed in this field to determine whether other factors impact on PVF and joint angulation on landing, including age/experience and breed/type of dogs and landing surface.

## Data Availability Statement

The raw data supporting the conclusions of this article will be made available by the authors, without undue reservation.

## Ethics Statement

The animal study was reviewed and approved by Nottingham Trent University, School of Animal, Rural and Environmental Sciences School Ethics Group (reference number ARE192042). Written informed consent was obtained from the owners for the participation of their animals in this study.

## Author Contributions

AC secured funding to support the research, conceptualized, designed, and managed the project. EW performed statistical analysis. All authors collected data, drafted, revised, read, and approved the submitted version of the manuscript.

## Funding

This project was funded by The Kennel Club, for which the authors wish to extend their gratitude.

## Conflict of Interest

JB was a member of The Kennel Club, Chair of The Kennel Club's Activities Health and Welfare Group and a member of The Kennel Club's Dog Health Group and consults on canine matters on an independent basis. The remaining authors declare that the research was conducted in the absence of any commercial or financial relationships that could be construed as a potential conflict of interest.

## Publisher's Note

All claims expressed in this article are solely those of the authors and do not necessarily represent those of their affiliated organizations, or those of the publisher, the editors and the reviewers. Any product that may be evaluated in this article, or claim that may be made by its manufacturer, is not guaranteed or endorsed by the publisher.

## References

[1] The Kennel Club. New to Working Trials? (2021). Available online at: https://www.thekennelclub.org.uk/events-and-activities/working-trials/new-to-working-trials/ (accessed October 6, 2021).

[2] The Kennel Club. Working Trial and Bloodhound Trial Regulations. (2021). Available online at: https://www.thekennelclub.org.uk/media/3469/working-trial-and-bloodhound-trial-i-regulations.pdf (accessed October 6, 2021).

[3] Levy M Hall C Trentacosta N Percival M. A preliminary retrospective survey of injuries occurring in dogs participating in canine agility. Vet Compar Orthopaedics Traumatol. (2009) 22:321–4. 10.3415/VCOT-08-09-0089 19597633

[4] Canapp SO. Shoulder conditions in agility dogs. Clean Run Focus Canine Sports Med. (2007) 7:1–5.

[5] Cullen KA Dickey JP Bent LR Thomason JJ Moens NMM. Internet based survey of the nature and perceived causes of injury to dogs participating in agility training and competition events. J Am Vet Med Assoc. (2013) 243:1010–8. 10.2460/javma.243.7.1010 24050568

[6] Cullen KA Dickey JP Bent LR Thomason JJ Moens NMM. Survey based analysis of risk factors for injury among dogs participating in agility training and competition events. J Am Vet Med Assoc. (2013) 243:101–24. 10.2460/javma.243.7.1019 24050569

[7] Birch E Carter A Boyd J. An examination of jump kinematics in dogs over increasing hurdle heights. Compar Exer Physiol. (2016) 12:91–8. 10.3920/CEP150037

[8] Birch E Boyd J Doyle G Pullen A. The effects of altered distances between obstacles on the jump kinematics and apparent joint angulations of large agility dogs. Vet J. (2015) 204:174–8. 10.1016/j.tvjl.2015.02.019 25841897

[9] Birch E Boyd J Doyle G Pullen. A. Small and medium agility dogs alter their kinematics when the distance between hurdles differs. Compar Exer Physiol. (2015) 11:75–8. 10.3920/CEP150015

[10] Yanoff SR Hulse DA Hogan HA Slater MR Longnecker MT. Measurements of vertical ground reaction force in jumping dogs. Vet Compar Orthopaedics Traumatol. (1992) 5:44–50. 10.1055/s-0038-163306626606158

[11] Powers PNR Harrison AJ. Models for biomechanical analysis of jumping horses. J Equine Vet Sci. (1999) 19:799–806. 10.1016/S0737-0806(99)80172-5

[12] Clayton HM Barlow DA. The effect of fence height and width on the limb placements of show jumping horses. J Equine Vet Sci. (1989) 9:179–85. 10.1016/S0737-0806(89)80046-2

[13] Hole SL Clayton HM Lanovaz JL. A note on the linear and temporal stride kinematics of Olympic show jumping horses between two fences. Appl Anim Behav Sci. (2002) 75:317–23. 10.1016/S0168-1591(01)00194-0

[14] Pfau T Garland de Rivaz A Brighton S Weller R. Kinetics of jump landing in agility dogs. Vet J. (2011) 190:278–83. 10.1016/j.tvjl.2010.10.008 21093327

[15] Pardey D Tabor G Oxley JA Wills AP. Peak forelimb ground reaction forces experienced by dogs jumping from a simulated car boot. Vet Rec. (2018) 182:716. 10.1136/vr.104788 29622683

[16] Wang M Song Y Valentin S Baker JS Gu Y. Kinetic analysis of felines landing from different heights. PeerJ. (2019) 12:e8007. 10.7717/peerj.8007 31737447 PMC6857581

[17] Feeney LC Lin CF Marcellin-Little DJ Tate AR Queen RM Yu B. Validation of two-dimensional kinematic analysis of walk and sit-to-stand motions in dogs. Am J Vet Res. (2007) 68:277–82 10.2460/ajvr.68.3.277 17331017

[18] R Core Team. R: A Language and Environment for Statistical Computing. Vienna: R Foundation for Statistical Computing (2020).

[19] Bates D Mächler M Bolker B Walker S. Fitting linear mixed-effects models using lme4. J Stat Softw. (2015) 67:1–48. 10.18637/jss.v067.i01

[20] Lenth RV. emmeans: Estimated Marginal Means, aka Least-Squares Means. R package version 1.5.4. (2021).

[21] Fox J Weisberg S. An R Companion to Applied Regression, Third Edition. Thousand Oaks CA: Sage (2019).

[22] Wickham H. Ggplot2: Elegant Graphics for Data Analysis. New York, NY: Springer Verlag (2016). 10.1007/978-3-319-24277-4

[23] Söhnel K Rode C de Lussanet M Wagner H Fischer MS Andrada E. Limb dynamics in agility jumps of beginner and advanced dogs. J Experi Biol. (2020) 223:jeb202119. 10.1242/jeb.202119 32098886

[24] Miró F López P Vilar JM Galisteo AM Vivo J Garrido-Castro JL. Comparative kinematic analysis of hurdle clearance technique in dogs: a preliminary report. Animals. (2020) 10:2405. 10.3390/ani10122405 33339144 PMC7765657

[25] Powers PNR Harrison AJ. A study on the techniques used by untrained horses during loose jumping. J Equine Vet Sci. (2000) 20:845–50. 10.1016/S0737-0806(00)80115-X

[26] Duggan BM Hocking PM Clements DN. Gait in ducks (Anas platyrhynchos) and chickens (Gallus gallus) similarities in adaptation to high growth rate. Biol Open. (2016) 5:1077–85. 10.1242/bio.018614 27387535 PMC5004611

[27] Voss K Wiestner T Galeandro L Hässig M Montavon MP. Effect of dog breed and body conformation on vertical ground reaction forces, impulses, stance times. Vet Comp Orthop Traumatol. (2011) 24:106–12. 10.3415/VCOT-10-06-0098 21243175

[28] Hernlund E Egenvall A Roepstroff L. Kinematic characteristics of hoof landing in jumping horses at elite level. Equine Vet J. (2010) 42:462–7. 10.1111/j.2042-3306.2010.00187.x 21059046

